# 3DSeqCheck: A web-based tool for verifying sequence consistency between a 3D structure file and the corresponding UniProt entry

**DOI:** 10.1016/j.jmb.2025.169620

**Published:** 2025-12-30

**Authors:** Anja Conev, Suhail A. Islam, Ifigenia Tsitsa, Alessia David, Michael J.E. Sternberg

**Affiliations:** 1 Centre for Integrative Systems Biology and Bioinformatics, Department of Life Sciences, Imperial College London, London SW7 2AZ, UK

## Abstract

UniProt is a central repository of protein sequences and annotations, with entries being updated several times a year as new sequencing evidence is collected. By contrast, protein structure resources often evolve at a different pace. The AlphaFold database remained unchanged for four years, until September 2025, during which time nearly 3% of the associated sequences underwent revisions in UniProt. In a range of bioinformatics tasks, protein structure data is paired with sequence annotations from UniProt. Mapping annotations to outdated structure files can lead to errors in downstream analysis. While this concern has been addressed for experimental structures, efforts for the modelled structures are lacking. 3DSeqCheck is a lightweight web tool that enables quick comparison of the sequence of modelled and experimental structures to the latest UniProt entries. 3DSeqCheck provides an interactive visual panel of the alignment and the comparison of the residue numbering and can be accessed freely at: https://missense3d.bc.ic.ac.uk/3dseqcheck and https://github.ic.ac.uk/ImperialCollegeLondon/check3Dseq

## Graphical abstract

**Figure:**
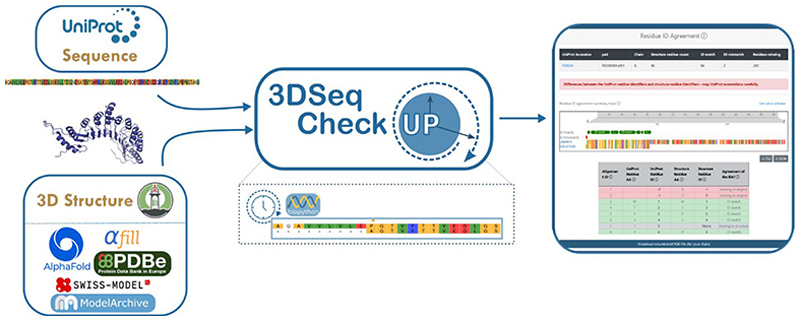

## 1 Introduction

DeepMind’s AlphaFold2 [1,2] models achieved outstanding accuracy in the CASP14 evaluation of protein structure prediction. In 2022, in collaboration with the European Institute of Bioinformatics (EBI), the AlphaFoldDB (AlphaFold DataBase) was released, providing over 200 million predicted structures [2]. Structure models of an unprecedented number of sequences are now available for researchers to study for a range of applications [3] including function prediction [4], the binding of small molecules [5], [6], understanding protein-protein interactions [7] and evaluating mutational effects [8]. Bioinformatics researchers are increasingly connecting their workflows to structural data such as AlphaFoldDB.

We have recently reported a discrepancy between AlphaFoldDB and UniProt that affected around 3% of all proteins in the human proteome, corresponding to 631 AlphaFoldDB entries that were found to be inconsistent with their related UniProt records in release 2025_03, published June 18, 2025 [9]. Out of the 631 inconsistent entries, 116 were made redundant by UniProt, 295 had deletions or insertions in the sequence since AlphaFoldDB deposition and 220 have the same sequence length but some variations in the sequence. The same issue persists across different species, with around 2.5% of the mouse proteome in AlphaFoldDB being inconsistent with the UniProt sequences. We highlighted the impact of sequence mismatch on the mapping of functional annotations, such as the location of transmembrane domains and missense variants.

In recent months, the synchronization of UniProt and AlphaFoldDB has been addressed on several levels. First, the UniProt [10] and SwissMODEL [11] platforms displayed warnings when users selected outdated AlphaFoldDB models on their platforms. Similarly, AlphaFoldDB at first provided warnings on its site. Finally, in September 2025, the AlphaFoldDB announced a new release of the models, synced with UniProt version 2025_03. While the latest release AlphaFoldDB provides up to date models, it is not clear how frequent releases will be in the future. Resources that were built on top of the old AlphaFoldDB models such as AlphaFill and AlphaMissense currently remain outdated. The need to keep the modelled structures up to date with UniProt entries extends beyond the AlphaFoldDB. 3D-Beacons [12] initiative, launched in 2022, allows users to fetch structure models corresponding to UniProt accession codes from various resources including the AlphaFoldDB [2], HegeLab.org [13], Isoform.io [14], ModelArchive [15], LevyLab [16], AlphaFill [17], SWISS-MODEL [11]. Many of these models may require checking synchronisation with UniProt before use.

Experimentally resolved structures have also been subject to the issue of integrating function annotations between the structure and the UniProt entries. For experimental structures, a substantial effort was undertaken by the community to provide such mappings. Structure Integration with Function, Taxonomy and Sequences (SIFTS) resource [11, 12, 13] has addressed this concern and has been maintained by UniProt, PDBe and EBI since 2002. SIFTS provides residue mappings such that other downstream services can renumber the residues in the structure according to the given UniProt entry. This is important because the residue identifier in a PDB file is simply a label assigned by the authors of the file and there is no requirement for it to correspond to the UniProt numbering and associated functional annotations. Other resources, such as PDBRenum [21], have been built to integrate SIFTS annotations and mappings. As part of their integrated sequence-structure viewer, the RCSB-PDB [22] shows the sequence from the structure aligned to the corresponding UniProt sequence using the SIFTS mappings. However, SIFTS infrastructure and the related tools only handle experimental structures.

**Figure 1:**
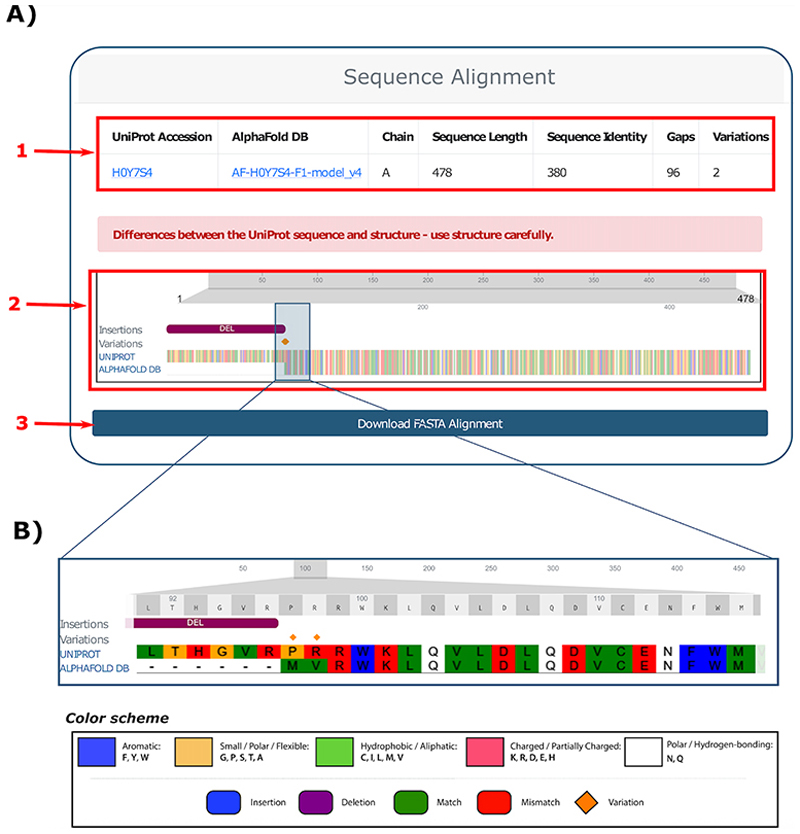
Check3DSeq Results Page - Sequence Alignment. A) Alignment results between the structure and UniProt sequences. (1) summary table; (2) alignment panel; (3) button for downloading the alignment in a FASTA format. B) Zoomed in perspective of the alignment segment between residue position 91 and residue position 116 in the UniProt sequence. The deleted region is shown with a purple round rectangle track above the sequences, while the variations are depicted in the orange diamond track. The color scheme for the sequence and the tracks is shown below the alignment.

**Figure 2:**
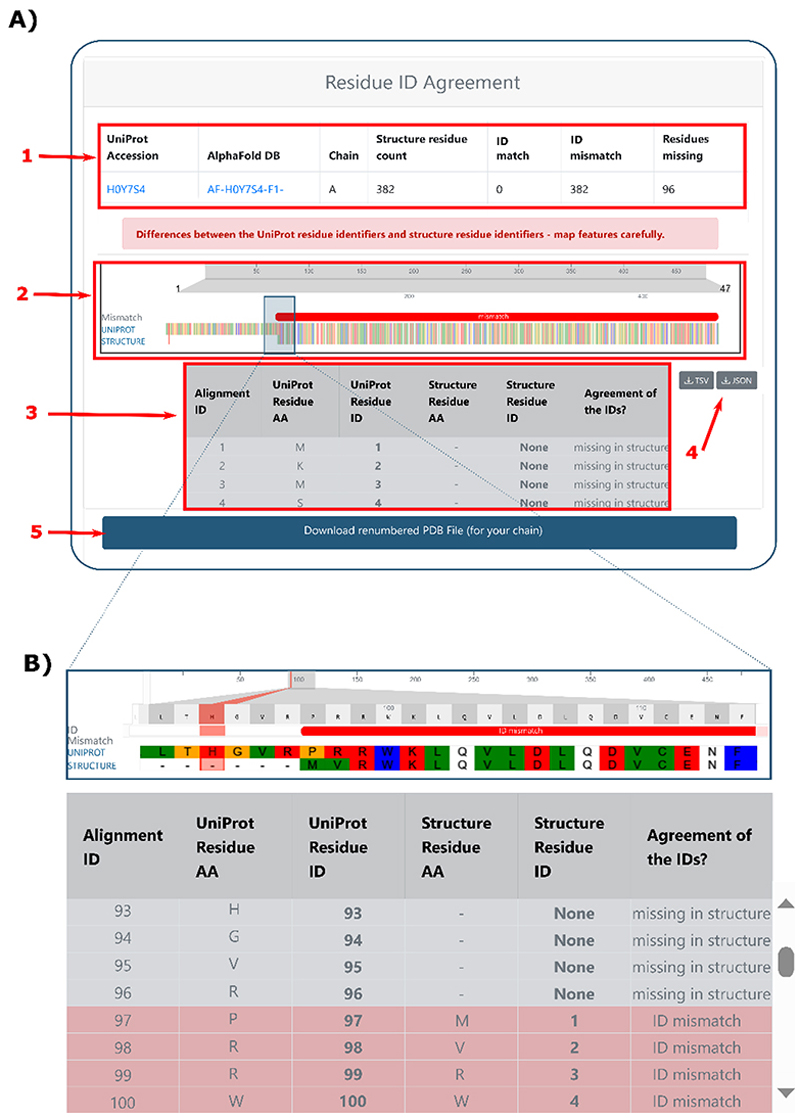
Check3DSeq Results Page - Residue Identifier Agreement. A) Residue ID comparison between the structure and UniProt sequences. (1) Summary table; (2) alignment panel; (3) detailed residue table; (4) buttons for downloading the results in both TSV and JSON formats. (5) Button for downloading the renumbered structure. B) Zoomed in perspective of the alignment segment between residue position 91 and residue position 114 in the UniProt sequence. Residues where the structure and UniProt IDs are different are marked in the red rectangle track above the sequences. Detailed residue table for residues 93-100 is displayed below the alignment and highlights residues where the IDs in UniProt and in the structure do not match.

In outline, 3DSeqCheck provides a simple entry. The user inputs the UniProt identifier for the protein whose sequence they wish to compare (Figure S1). They can compare models from different structure databases or upload their own structure file. The results page of 3DSeqCheck shows the alignment of the UniProt sequence to that extracted from the structure. It provides details of the alignment on two levels. First, it provides a detailed view of the sequence alignment between the UniProt sequence and structure (Figure 1). Regions that are missing or added in structure, as well as regions that are different in the two sequences, are highlighted and an overall match percentage is provided. Second, it compares the residue identifiers inside the structure to those in the UniProt sequence (Figure 2). Each aligned residue is presented in a table with columns containing UniProt identifiers as well as the residue identifiers of the structure. Users are presented with the overall percentage of residues that match in identifiers, and they can download a renumbered file as well as the results in both TSV and JSON formats.

3DSeqCheck can be accessed at https://missense3d.bc.ic.ac.uk/3dseqcheck, where it ensures reliable sequence–structure correspondence across diverse modelling resources, helping researchers avoid annotation errors and make more confident use of structural data in their analyses. In addition, the source code is available for download on GitHub at https://github.ic.ac.uk/ImperialCollegeLondon/check3Dseq.

## 2 3DSeqCheck user pipeline

### Input description

The 3DSeqCheck submission form is displayed on the main page for users to interact with the app (Figure S1). Here, the user is prompted to input the data for the structure they want to validate.

UniProt ID is the first required input to 3DSeqCheck. The UniProt ID should correspond to the entry that your structure describes, it is used to fetch the most up-to-date sequence that is used as a *reference sequence* in the 3DSeqCheck. Structure Source is the second required input to 3DSeqCheck. The Structure Source should correspond to the structure the user wants to validate. The *query sequence* will be extracted from this given structure. There are currently two supported ways to supply a structure:

- *From a Database -* a structure will be fetched from the corresponding database (options include: AlphaFoldDB, AlphaFill, ModelArchive, Isoform.io, Protein Ensemble Database, HegeLab, LevyLab, PDBe, SWISS-MODEL) using the 3DBeacons API [12].
- *Custom PDB File* - by choosing a custom file as source, the uploaded file will be used as input. In addition to the PDB file, the user needs to provide the chain ID for the chain corresponding to the supplied UniProt ID.

Note that 3DSeqCheck imposes limits to the input size to prevent excessive computational overhead. Namely, custom PDB files are restricted to a maximum size of 5 MB. In addition, AlphaFoldDB entries containing more than 2,700 amino acids are not supported for comparison as they are split across multiple files and are not unavailable through the AlphaFoldDB API. Users are presented with a warning message if their inputs exceed these limits.

### Extracting the sequences

To obtain the up-to-date *reference sequence*, 3DSeqCheck queries the UniProt REST API[23] using the input UniProt ID. The *sequence of the query structure* is obtained by extracting it from the structure source by identifying the residues that are in the `ATOM` section of the structure file (with the matching chain ID, in the case of custom file input).

### Alignment

The pairwise sequence alignment of the reference and query sequences is performed using the implementation of the Needleman-Wunsch algorithm provided in BioPython’s Align module [24] with the default parameters.

### Output visualization

Upon submission of the input form, the user is presented with a Results page which displays : 1) a concise overview of the alignment between the reference sequence and the amino acid sequence derived from the structure file (Figure 1), and 2) a detailed residue-level analysis, tabulating the type and extent of discrepancies identified (Figure 2).

The results of the concise analysis are displayed at the top of the Results page in a summary table (Figure 1A) which presents:

- length of aligned sequences
- sequence identity between the query sequence (structure) and the reference sequence (UniProt)
- number of gaps (insertions/deletions to the reference UniProt sequence as compared to the query sequence) in the alignment
- number of variations (different amino acids between the reference UniProt sequence and the query sequence) in the alignment

The match assessment message is displayed below the summary table and contains two possible outcomes:

- *Your structure matches the UniProt sequence, safe to use!* is displayed when the sequence identity is 100% and the UniProt sequence fully matches the sequence in the structure. This corresponds to a perfect alignment, and the recommendation is to freely use the UniProt annotations for the supplied UniProt ID on this structure.
- *Differences between the UniProt sequence and structure - use structure carefully*. is displayed in case the sequence identity is less than 100%. This corresponds to an alignment of varying quality due, for example, to the presence of gaps, insertions or residue mismatch. The recommendation is to use the UniProt annotations carefully for this structure.

The alignment is presented in an interactive dashboard using the Nightingale web components [25], and the insertions/deletions/variations are displayed in separate tracks on top of the alignment (Figure 1B). FASTA file contents are available for download.

The second section of the Results page presents the detailed analysis of the agreement between the identifiers of residues in the structure and the UniProt identifiers (Figure 2A). Structure residue identifiers are contained in columns 23 to 26 of the ATOM lines of the PDB file. These identifiers, along with the chain identifiers are used to identify parts of the structure that the user is interested in. UniProt residue identifiers are associated with the position of the residue in the UniProt sequence (e.g., the first residue has the identifier 1, the tenth residue identifier 10 etc.). In the second section of the Results page, the agreement between these identifiers is inspected in detail. The following information is included in a summary table:

- number of residues in the structure
- number of residues in the structure for which the identifiers match the UniProt sequence identifiers
- number of residues in the structure for which the identifiers do not match the UniProt sequence identifiers
- number of residues that are missing from the structure and are present in the UniProt sequence

The match assessment message is displayed below the summary table and contains two possible assessments:

- *Your structure matches the UniProt sequence identifiers, safe to map features!* is displayed in case all the residue identifiers of the structure match all the identifiers of the UniProt sequence. This corresponds to a perfect identifier match, and the recommendation is to freely use the UniProt annotations for those residues that are not missing in the structure.
- *Differences between the UniProt residue identifiers and structure residue identifiers - map UniProt annotations carefully*. is displayed in case the match between identifiers is not perfect. The recommendation is to use the UniProt annotations carefully for the supplied UniProt ID on this structure.

ID match/mismatch labels are also visually displayed in separate tracks on top of the alignment in the interactive dashboard (Figure 2B).

The detailed residue table (Figure 2B) shows the following information for each residue in the alignment result:

- Alignment ID - residue identifier in the alignment (starting from 1)
- UniProt residue AA - amino acid at that position of the UniProt sequence
- UniProt residue ID - identifier in the UniProt sequence (starting from 1 of the first residue in the UniProt sequence)
- Structure residue AA - amino acid on that position in the structure file
- Structure residue ID - identifier in the structure file (extracted from the ATOM lines of the structure file)
- label - *ID match*/*ID mismatch*/*missing*

Insertions are defined as residues present in the experimental structure but absent from the UniProt reference sequence, while deletions correspond to UniProt residues missing from the structure. Inserted residues are excluded from the renumbered structure to preserve a one-to-one mapping with UniProt, and users are warned when this results in the removal of residues from the original structure. The table with residue mappings is available for download in TSV and JSON formats. The renumbered structure file for the selected chain is available for download in PDB format.

## 3 Example

### PRAME family member 26 (UniProt ID: H0Y7S4)

PRAME (PReferentially expressed Antigen of MElanoma) is a protein family of leucine-rich repeat (LRR) proteins expressed in several cancer cells and recognized by cytolytic T lymphocytes [26]. PRAMEF26, UniProt entry H0Y7S4, is a well characterised member of the PRAME family, and represented in AlphaFill by an outdated sequence model. Running entry H0Y7S4 through the 3DSeqCheck, we observe that the current UniProt entry contains 478 amino acids, while the AlphaFill structure contains 382 with a region of 96 consecutive amino acids deleted in the C-terminus of the structure, with respect to the current UniProt entry (Figure 1A). In addition, there are two amino acids at the beginning of the AlphaFill model that differ from the UniProt sequence (Figure 1B). Due to the deletion at the start of the sequence, the residue identifiers in the AlphaFill structure (Figure 2A) are in complete disagreement with the UniProt identifiers.

**Figure 3:**
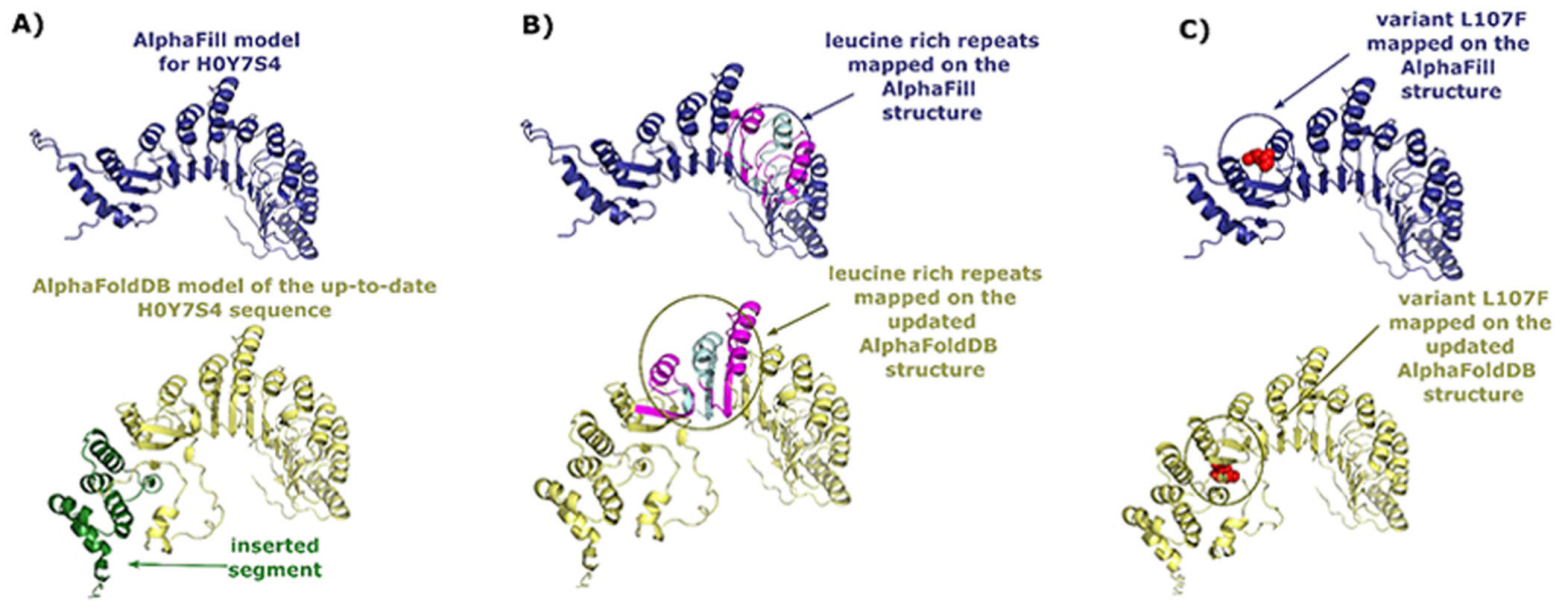
*PRAMEF26* (H0Y7S4) example. A) AlphaFill model (blue) and up-to-date AlphaFoldDB model (yellow). The inserted segment is highlighted in green. B) AlphaFill and up-to-date AlphaFoldDB models with LRR domains highlighted. C) AlphaFill and up-to-date AlphaFoldDB models with the L107F variant highlighted.

In this example, we showcase the problems users may encounter when using an outdated AlphaFill structure in a widely used molecular graphics program such as PyMol [27]. We compare the AlphaFill model with the up-to-date AlphaFoldDB model of the sequence corresponding to the latest UniProt entry (Figure 3A). The recent UniProt sequence update introduces an additional alpha-helical domain at the N terminus of PRAMEF26 structure (Figure 3A, highlighted green). Furthermore, the deletion causes the sequence annotations to be shifted by 96 amino acids (Figure 2B). This shift affects the annotation of LLR (Figure 3C) domains as well as the numbering of the leucine at position 107, which harbours the amino acid substitution p.L107F. In the out-of-date AlphaFill model, leucine 107 corresponds to position 11 and position 107 is occupied by a methionine (Figure 3D). Discrepancy in the AlphaFill model and the UniProt sequence would go unnoticed by molecular graphics programs such as PyMol[27] or Chimera[28] in which the structural features (i.e., variants, domains, binding sites) are easily highlighted. Furthermore, it can lead to inaccuracies when using downstream structure-based algorithms such as Missense3D [8], potentially misrepresenting the functional or structural impacts of variants. Addressing these issues is critical for maximising the utility of structure models and ensuring their integration into downstream tools and resources.

## 4 Conclusion

3DSeqCheck is a web application designed for rapid and easy validation of the consistency of a protein structure against the latest UniProt entries. The interactive sequence alignment overview and residue-by-residue identifier comparison present 3DSeqCheck as a resource for the community using 3D structures in downstream analyses.

With the example of PRAMEF26 protein (UniProt entry H0Y7S4) we show how discrepancies between UniProt sequences and corresponding protein structures pose risks on two levels. First, a structure file may contain a different sequence, or only a fragment of the query UniProt sequence. Users may draw wrong conclusions about the protein, as structural domains could be inaccurately represented or incompletely modelled. Second, even when the sequences are in agreement, there is a risk that residue identifiers used in the structure file do not agree with the identifiers within the UniProt sequence. Users risk mapping annotations from UniProt to the wrong portions of the structure. Check3DSeq allows users to interactively inspect such residue identifier disagreement in the bottom panel of the results page (Figure 2).

The potential discrepancies between a structural file and UniProt residue identifiers could result in errors when using molecular graphics programs such as PyMOL[27] and Chimera[28] where residue identifiers are used to locate and highlight positions of particular features (e.g., variants, binding sites, post-translational modifications). 3DSeqCheck is useful in these cases as it allows users to download a renumbered structure file that corresponds to the UniProt sequence.

3DSeqCheck highlights and explains the underlying differences between the modelled structure and the UniProt entry. Nevertheless, correct mapping remains critical—for example, when investigating the structural context of specific variants, since even small inconsistencies can compromise biological interpretation. While the latest release of AlphaFoldDB provides up-to-date models, it is not clear how frequent these updates will be in the future; other sources of models accessible through 3DBeacons, such as AlphaFill, ModelArchive and Isoform.io, suffer from the same syncronisation issue. 3DSeqCheck can be applied to a structure file from any source, thereby offering broader utility across modelling resources and ensuring greater reliability in the integration of structural data with sequence-based annotations, and thus it facilitates a more accurate use of protein structure data.

As protein structure modelling becomes more accurate, the models are increasingly being integrated into a variety of different bioinformatics pipelines. To ensure the reliability of downstream analyses, it is essential that modelled structures remain consistent with major reference databases and tools [9]. 3DSeqCheck is a contribution to the software ecosystem allowing for robust integration of protein structure data with functional annotations.

## Supplementary Material

SI

## Highlights

- UniProt protein entries are updated regularly as new sequencing evidence emerges.
- Protein structure files often lag behind these sequence updates.
- Mapping UniProt annotations to outdated structures can hinder downstream analysis.
- 3DSeqCheck aligns sequences and checks structures against the latest UniProt entry.
- 3DSeqCheck provides a visual overview of structure consistency with UniProt.
