# Supplementary material for: 3DSeqCheck: A web-based tool for verifying sequence consistency between a 3D structure file and the corresponding UniProt entry
Source: J Mol Biol. Author manuscript; Available in PMC 2026 Sep 1. (PMC7618724; doi:10.1016/j.jmb.2025.169620)
Supplement: Supplementary file 1 [file EMS212329-supplement-SI.pdf]

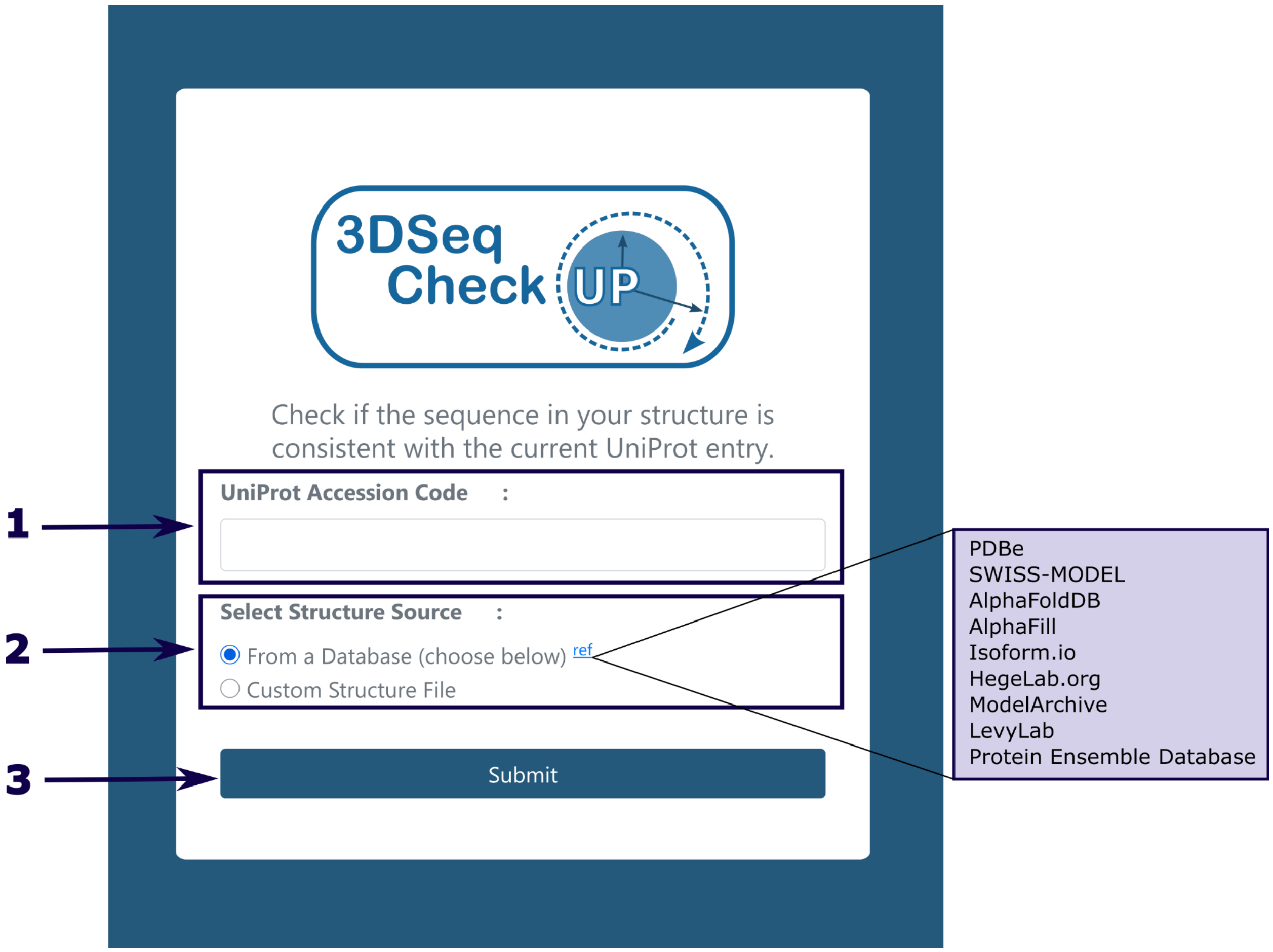

Figure S1. Check3DSeq Input Form. (1) required text input UniProt identifier; (2) required structure input radio button for either Database entry or PDB file upload and a chain identifier; (3) submission button for running the Check3DSeq validation.
